# The role of furin cleavage site in SARS-CoV-2 spike protein-mediated membrane fusion in the presence or absence of trypsin

**DOI:** 10.1038/s41392-020-0184-0

**Published:** 2020-06-12

**Authors:** Shuai Xia, Qiaoshuai Lan, Shan Su, Xinling Wang, Wei Xu, Zezhong Liu, Yun Zhu, Qian Wang, Lu Lu, Shibo Jiang

**Affiliations:** 1 Key Laboratory of Medical Molecular Virology (MOE/NHC/CAMS), School of Basic Medical Sciences, Fudan University, 130 Dong An Road, Shanghai, 200032 China; 2 National Laboratory of Biomacromolecules, Institute of Biophysics, Chinese Academy of Sciences, Beijing, 100101 China

**Keywords:** Pathogenesis, Infection

**Dear Editor**,

The rapid spread of SARS-CoV-2 (also known as 2019-nCoV and HCoV-19^1^), a novel lineage B betacoronavirus (βCoV), has caused a global pandemic of coronavirus disease (COVID-19). It has been speculated that RRAR, a unique furin-like cleavage site (FCS) in the spike protein (S), which is absent in other lineage B βCoVs, such as SARS-CoV, is responsible for its high infectivity and transmissibility.^2^

A coronavirus (CoV) infects the target cell by either cytoplasmic or endosomal membrane fusion. No matter what path it chooses, the final step of viral entry involves the release of RNA into the cytoplasm for replication. Therefore, the fusion capacity of the CoV-S is a leading indicator of infectivity of the corresponding virus. Consisting of S1 receptor-binding subunit and S2 fusion subunit, CoV-S needs to be primed through cleavage at S1/S2 site and S2′ site in order to mediate the membrane fusion (Fig. 1a). Previous studies have shown that an insertion of FCS consisting of multiple basic amino acids in the cleavage site of the haemagglutinin (HA) is associated with high virulence of influenza viruses.^3^ Coincidentally, phylogenetic analysis of SARS-CoV-2 identified an insertion of RRAR (FCS) at the S1/S2 site of SARS-CoV-2-S, which is absent in SARS-CoV and other SARS-related coronaviruses (SARSr-CoVs), particularly RaTG13, which has 96% identity of its genomic sequence to that of SARS-CoV-2 (Fig. 1b and Supplementary Fig. S1). Therefore, it has been speculated that this unique FCS may provide a gain-of-function, making SARS-CoV-2 easily enter into the host cell for infection, thus efficiently spreading throughout the human population, compared to other lineage B betacoronaviruses.^2^

**Fig. 1:**
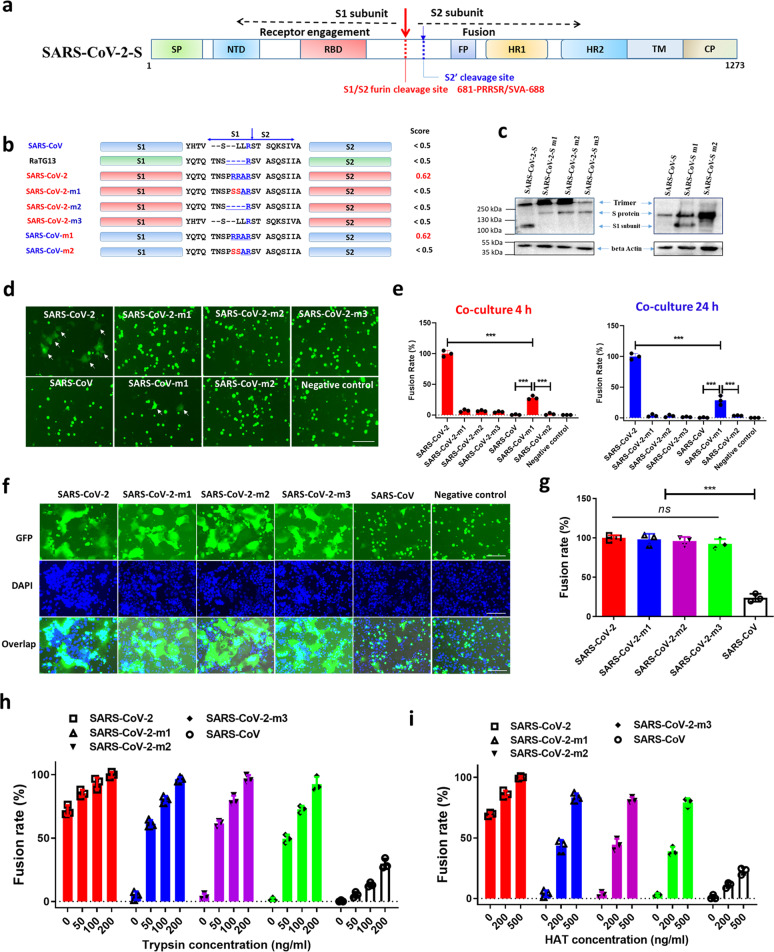
The function of furin cleavage site in SARS-CoV-2-S mediated fusion. **a** Schematic representation of SARS-CoV-2 S protein and the location of S1/S2 and S2′ cleavage site. SP, signal peptide; FP, fusion peptide; HR, heptad repeat domain; TM, transmembrane domain; CP, cytoplasmic domain. **b** Mutated SARS-CoV-2 S proteins with mutation in S1/S2 region, including SARS-CoV-2-m1 (mutating “RRAR” into “SSAR”), SARS-CoV-2-m2 (deleting four amino acids, “PRRA”), SARS-CoV-2-m3 (replacing “QTQTNSPRRARSVASQSII” in SARS-CoV-2 with “HTVSLLRSTSQKSIV” derived from SARS-CoV), SARS-CoV-m1 (replacing “HTVSLLRSTSQKSIV” in SARS-CoV with “QTQTNSPRRARSVASQSII” derived from SARS-CoV-2), and SARS-CoV-m2 (mutating “RRAR” in SARS-CoV-m1 into “SSAR”). Prediction scores for the S1/S2 furin cleavage site in S protein were analyzed by using the ProP 1.0 server (www.cbs.dtu.dk/services/ProP/). **c** Western blot analysis of S protein expression in 293T cells. **d** Representative images of cell–cell fusion between 293T/SARS-CoV-2/EGFP, 293T/SARS-CoV-2-m1/EGFP, 293T/SARS-CoV-2-m2/EGFP, 293T/SARS-CoV-2-m3/EGFP, 293T/SARS-CoV/EGFP, 293T/SARS-CoV-m1/EGFP, and 293T/SARS-CoV-m2/EGFP effector cells and target cells (Huh-7), using the fusion between target cells and 293T/EGFP effector cells without S-expression as negative control. Representative fused cells are indicated by white arrows. Scale bar = 400 µm. **e** Statistical analysis of fusion rates mediated by wild-type or mutated S protein after co-culture for 4 h (left) or 24 h (right), taking the fusion rate of SARS-CoV-2 group as 100%. **f** Representative images of cell–cell fusion between target and effector cells with indicated S protein in the presence of trypsin (200 ng/ml). Scale bar = 400 µm. **g** Statistical analysis for (**f**), taking the fusion rate of the SARS-CoV-2 group as 100%. **h**, **i** Fusion rates of cell–cell fusion between target cells and series of effector cells in the presence of indicated concentration of trypsin (**h**) or HAT (**i**), taking the fusion rate of SARS-CoV-2 group treated by trypsin (200 ng/ml) or HAT (500 ng/ml) as 100%. Experiments were repeated twice, and the data are expressed as means ± SD (error bar). Asterisks indicate significant differences (***P < 0.001); ns: no significance

There has been no report to prove this hypothesis experimentally so far. Therefore, we herein first analyzed the potential role of this FCS in CoV-S-mediated fusion via an S-mediated cell–cell fusion assay (Supplementary Fig. S2a). In this widely adopted cell–cell fusion system,^4^ CoV-S and green fluorescent protein gene were transferred into 293T cells. To mimic the viral fusion process, CoV-S on the transduced 293T cell as the effector cell could mediate its fusion of the effector cell with the receptor-bearing cell as the target cell. Then, by calculating the ratio of the fused cells, we can assess the fusogenic capacity of the S protein in the presence or absence of exogenous trypsin or human airway trypsin-like protease (HAT).

First, we constructed four vectors expressing SARS-CoV-2-S and its variants, SARS-CoV-2-m1, SARS-CoV-2-m2, and SARS-CoV-2-m3, as well as SARS-CoV-S and its variants, SARS-CoV-m1 and SARS-CoV-m2 (Fig. 1b). In SARS-CoV-2-S-m1, 682-RRAR-685 was replaced by 682-SSAR-685, while in SARS-CoV-2-S-m2 and m3, the hinge region between S1 and S2 of SARS-CoV-2 was replaced by that of RaTG13 and SARS-CoV, respectively. In the SARS-CoV-m1 and SARS-CoV-m2, the hinge region between S1 and S2 of SARS-CoV was replaced by that of SARS-CoV-2 and SARS-CoV-2-m1, respectively (Fig. 1b).

We then assessed the expression of the SARS-CoV-2-S and variants in 293T. Western blot analysis of 293T cells transduced with these vectors revealed that SARS-CoV-2-S, SARS-CoV-S and their variants were robustly expressed whereas only SARS-CoV-2-S and SARS-CoV-S-m1 that contain the FCS were processed in the biosynthesis process (Fig. 1c), confirming that FCS indeed works in SARS-CoV-2-S and SARS-CoV-S-m1.

Next, we compared the fusogenic capacity of SARS-CoV-2-S, SARS-CoV-S and their mutants via an S-mediated cell–cell fusion assay in the absence of exogenous trypsin or human airway trypsin-like protease (HAT). The transduced 293T cells were co-incubated with the target cells, Huh-7. After co-incubation for 4 h, 293T cells with wild-type SARS-CoV-2-S fused with the target cells, while cells remained unfused in the SARS-CoV-2-S-m1, m2, and m3 groups (Fig. 1d). Even after a 24-h incubation, the 293T cells with mutated SARS-CoV-2-S still remained unfused (Fig. 1e). Consistently, 293T/ACE2 cells transiently expressing SARS-CoV-2-S or SARS-CoV-S-m1 could naturally fuse with each other, while 293T/ACE2 cells bearing SARS-CoV-2-m1, SARS-CoV-2-m2, SARS-CoV-2-m3, SARS-CoV-S, or SARS-CoV-m2 could not (Supplementary Fig. S2b). Interestingly, SARS-CoV-2 or SARS-CoV-S-m1 that carries FCS could effectively mediate the cell–cell fusion, while others having no FCS could not (Fig. 1d and Supplementary Fig. S2b). These results suggest that FCS may play a role in CoV-S-mediated membrane fusion in the absence of trypsin or HAT.

Subsequently, we compared the fusogenic capacity of SARS-CoV-2-S and its mutants in the presence of trypsin. Surprisingly, like the FCS-containing SARS-CoV-2-S, all the mutants without functional FCS and SARS-CoV-S could effectively mediate cell–cell fusion in a trypsin concentration-dependent manner, although the SARS-CoV-S-mediated fusion activity is much lower than that of SARS-CoV-2-S (Fig. 1f–h). Similarly, treatment with HATs also effectively rescued the fusogenic capacity of SARS-CoV-2 without FCS in a dose-dependent manner (Fig. 1i).

These results suggest that the FCS may be not as critical as previously thought for the high fusion capacity of SARS-CoV-2 in an environment in the presence of HATs, such as human airway. Notably, only high concentration of trypsin could entirely recover the fusogenic capacity of SARS-CoV-2-S without FCS. Therefore, the conclusion of whether SARS-CoV-2 without FCS can still infect target cell with such high efficiency in human airway needs more in vitro and in vivo evidence. Besides, even though the introduction of RRAR-based FCS endowed SARS-CoV-S with the ability to mediate cell–cell fusion, the fusion rate is still significantly lower than that of SARS-CoV-2-S. Previous studies have shown that receptor binding domain (RBD) of SARS-CoV-2-S1 subunit had approximately 10- to 20-fold higher affinity to ACE2 than SARS-CoV RBD.^5^ Besides, fusion core structure formed by HR1 and HR2 domain in S2 subunit of SARS-CoV-2 is highly stable.^4^ Therefore, it can be inferred that the structure and characteristics of S protein may be responsible for the potent fusogenic activity of SARS-CoV-2-S, rather than the presence of FCS. Nevertheless, more studies on the COVID-19 animal models are necessary to clarify the exact role of the FCS or host-furin protease in SARS-CoV-2 infection.

## Supplementary information

Supplemental Material
