# Supplementary material for: The role of furin cleavage site in SARS-CoV-2 spike protein-mediated membrane fusion in the presence or absence of trypsin
Source: Signal Transduct Target Ther. 2020 Jun 12;5:92. doi: 10.1038/s41392-020-0184-0 (PMC7289711; doi:10.1038/s41392-020-0184-0)
Supplement: Supplementary file 1 — Supplemental Material [file 41392_2020_184_MOESM1_ESM.docx]

**Supplemental Material for**

**The role of furin cleavage site in SARS-CoV-2 spike protein-mediated membrane fusion in the presence or absence of trypsin**

Shuai Xia^1,†^, Qiaoshuai Lan^1,†^, Shan Su^1,†^, Xinling Wang^1,†^, Wei Xu^1^, Zezhong Liu^1^, Yun Zhu^2^, Qian Wang^1,^ *, and Lu Lu^1,^ *, Shibo Jiang^1,^ *

*** Correspondence:**

Lu Lu (lul@fudan.edu.cn), Shibo Jiang ([shibojiang@fudan.edu.cn), Qian](mailto:shibojiang@fudan.edu.cn,%20Qian) Wang (wang_qian@fudan.edu.cn).

^†^ These authors contributed equally: Shuai Xia, Qiaoshuai Lan, Shan Su, Xinling Wang

**Supplementary information and Figures**

Fig. S1 Phylogenetic analysis of SARS-CoV-2 spike protein.

Fig. S2 The cell-cell fusion mediated by S protein.

**
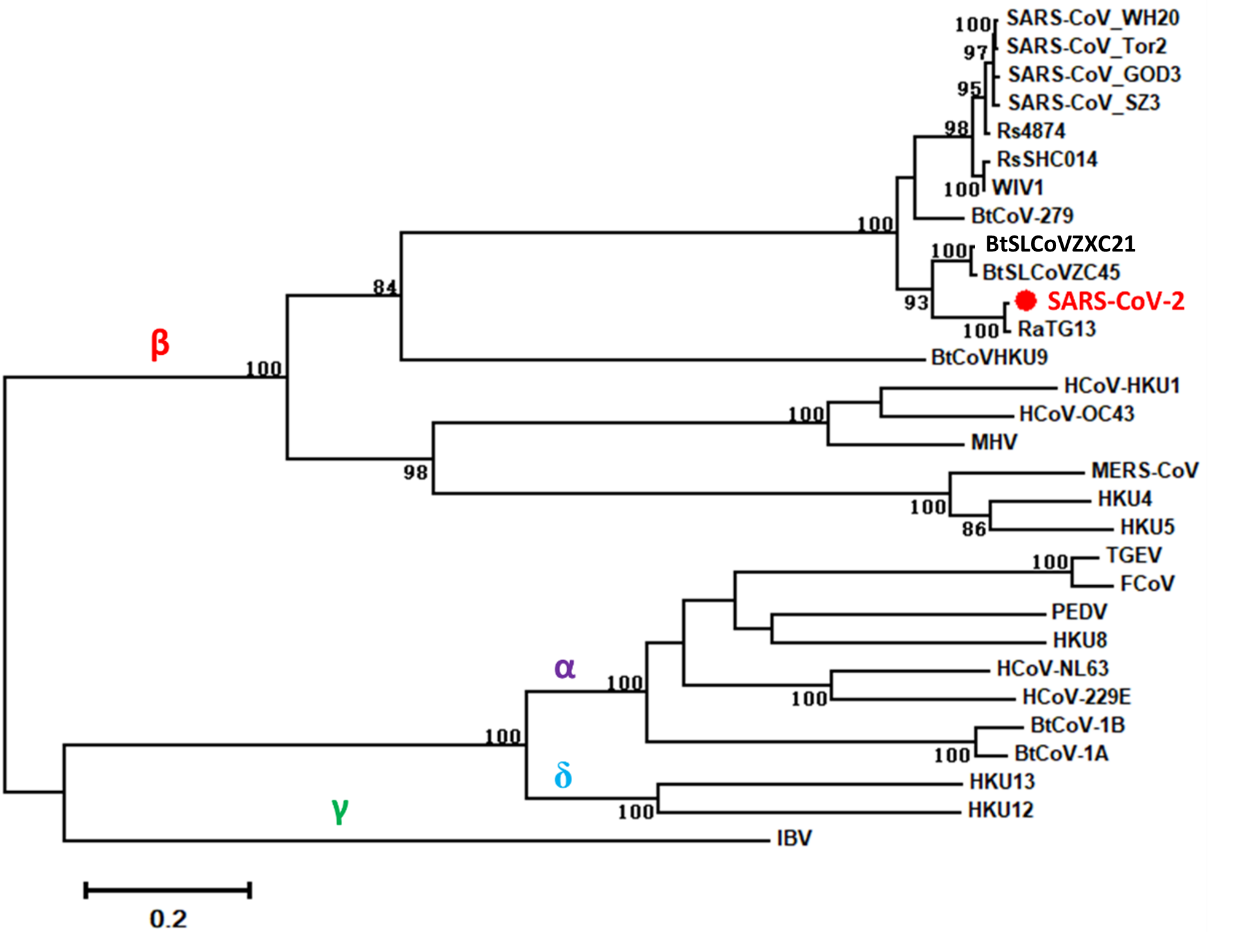
**

**Fig. S1 Phylogenetic analysis of SARS-CoV-2 spike protein.**

**
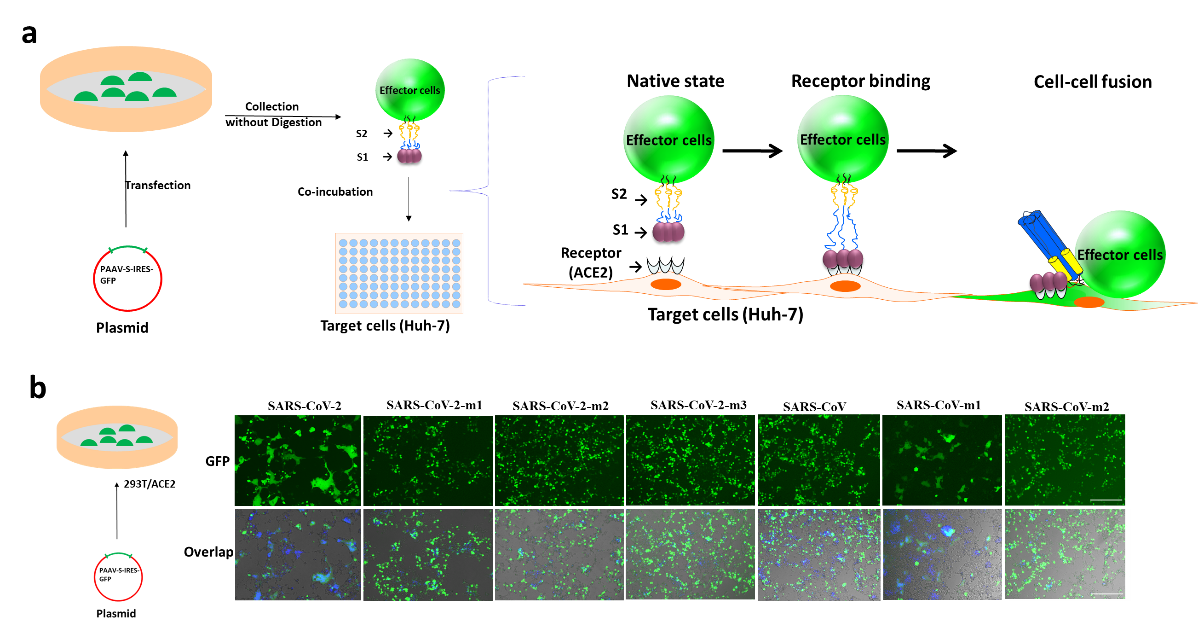
**

**Fig. S2 The cell-cell fusion mediated by S protein**

**a.** Schematic representation of S-mediated cell-cell fusion. 293T cells were transfected with plasmid pAAV-S-IRES-GFP to naturally present the viral S protein on cellular membrane surface and EGFP in cellular cytoplasm (293T/S/GFP). Huh-7 cells, naturally expressing the human ACE2 receptor, were used as target cells. **b.** Representative images of cell-cell fusion on 293T/ACE2 cells co-expressing hACE2 and S protein, 36 hours post-transfection, scale bar = 400 µm.

**Materials and methods**

***Cells and Plasmids***

Cell line 293T was obtained from ATCC (Manassas, VA, USA); the Huh-7 cell line was from the Chinese Academy of Science Cell Bank (Shanghai, China). All cell lines were cultured in Dulbecco’s Modified Eagle’s Medium (DMEM) with 10% fetal bovine serum (FBS). Plasmids: pAAV-SARS-CoV-2-S-IRES-EGFP, pAAV-SARS-CoV-2-S-m1-IRES-EGFP, pAAV-SARS-CoV-2-S-m2-IRES-EGFP, pC-DNA-3.1-SARS-CoV-2-S, pC-DNA-3.1-SARS-CoV-2-S-m1, pC-DNA-3.1-SARS-CoV-2-S-m2, pNL4-3.Luc.R-E and pAAV-IRES-EGFP were synthesized or preserved in our laboratory.

***Coronavirus phylogenetic analysis***

The full-length amino acid sequences of the spike of representative CoVs were downloaded from Genbank: PEDV (AHZ94887.1), HCoV-229E (AOG74783.1), HCoV-NL63 (AFV53148.1), Bt-CoV-1A (YP_001718605.1), Bt-CoV-1B (ACA52157.1), MHV (AAR92028.1), IBV (AKN20490.1), HCoV-OC43 (QDH43762,1), HCoV-HKU1 (AGW27881.1), BtCoV-HKU4 (YP_001039953.1), BtCoV-HKU5 (YP_001039962.1), MERS-CoV (MH454272.1), SARS-CoV_Tor2 (AY274119), SARS-CoV_GD03T13 (AY525636), SARS-CoV_SZ3 (AY304486), SARS-CoV_WH20 (AY772062.1), BtSCoV-SHC014 (AGZ48806.1), BtSCoV-Rs3367 (AGZ48818.1), BtSCoV-WIV1 (AGZ48828.1), HKU8 (NC_010438.1), HKU9 (HM211098.1), HKU12 (NC_011549.1), HKU13 (NC_011550.1), BtCoV-279 (ABG47069.1), BtSL-CoVZXC21 (MG772934.1), BtSL-CoVZC45 ( MG772933.1), RaTG13 ( MN996532.1) and SARS-CoV-2 (MN908947). Phylogenetic trees were constructed using MEGA6.06, and maximum likelihood estimation was performed using 100 bootstraps. The scale bar represents residue substitutions with only bootstrap support above 80% labeled.

***Cell-cell fusion assays***

1. In the absence of exogenous trypsin or HAT

Plasmid pAAV-IRES-S-EGFP, encoding S protein and EGFP, was transfected into 293T effector cells (293T/S/GFP). Huh-7 cells, naturally expressing human ACE2 receptors on the membrane surface, were used as target cells. 293T cells, transfected with plasmid pAAV-IRES-EGFP (293T/EGFP), were used as negative control. The effector cells (293T/S/GFP) were collected and resuspended. The free effector cells were added into target cells (Huh-7 cells), co-cultured in DMEM containing 10% FBS, 37 °C, for 4 or 24 h, then observed the fusion under the fluorescence microscope. Alternatively, transfected pAAV-IRES-EGFP or pAAV-S-IRES-EGFP into 293T/ACE2 cells, which constantly expressed ACE2 receptor. After 36 h, directly observed the fusion under the fluorescence microscope.

2. In presence of exogenous trypsin or HAT

The effector cells (293T/S/GFP) were added into target cells (Huh-7 cells) for coincubation in the presence of TPCK-trypsin or HATs with indicated concentration for 12 h, 37 °C, and then observed under fluorescence microscopy.

***Western blot***

Western blot was performed to detect the cleaved or uncleaved S protein in effector cells. Briefly, cell lysates were respectively separated by 10% tris-glycine gel, then transferred gel to PVDF membrane. After blocking with 5% non-fat milk in PBST 12 h at 4°C, the blots were respectively incubated with anti-S1 of SARS–CoV (Sino Biological, 40150-T62-COV2, 1:3000), which can crossly recognize SARS-CoV-2 S1 in Western blot assay, for 1 h at room temperature. After three washes, the blots were incubated with HRP-conjugated anti-rabbit IgG (1:5000) for 1 h at room temperature, and then imaged with Western blot substrate reagents under a Chemiluminescent Imaging System (Tanon-4600). Mouse anti-β-actin antibody (Proteintech, 66009-1-Ig, 1:3000) was used.
